# Deep Learning Combined with Quantitative Structure‒Activity Relationship Accelerates *De Novo* Design of Antifungal Peptides

**DOI:** 10.1002/advs.202412488

**Published:** 2025-02-08

**Authors:** Kedong Yin, Ruifang Li, Shaojie Zhang, Yiqing Sun, Liang Huang, Mengwan Jiang, Degang Xu, Wen Xu

**Affiliations:** ^1^ Zhengzhou Key Laboratory of Functional Molecules for Biomedical Research Henan University of Technology Zhengzhou Henan 450001 P. R. China; ^2^ College of Information Science and Engineering Henan University of Technology Zhengzhou Henan 450001 P. R. China; ^3^ School of Biological Engineering Henan University of Technology Zhengzhou Henan 450001 P. R. China; ^4^ School of Artificial Intelligence and Big Data Henan University of Technology Zhengzhou Henan 450001 P. R. China; ^5^ Law College Henan University of Technology Zhengzhou Henan 450001 P. R. China

**Keywords:** antifungal peptide, *de novo* design, deep learning, protein language model, QSAR

## Abstract

Novel antifungal drugs that evade resistance are urgently needed for *Candida* infections. Antifungal peptides (AFPs) are potential candidates due to their specific mechanism of action, which makes them less prone to developing drug resistance. An AFP *de novo* design method, Deep Learning‐Quantitative Structure‒Activity Relationship Empirical Screening (DL‐QSARES), is developed by integrating deep learning and quantitative structure‒activity relationship  empirical screening. After generating candidate AFPs (c_AFPs) through the recombination of dominant amino acids and dipeptide compositions, natural language processing models are utilized and quantitative structure‒activity relationship (QSAR) approaches based on physicochemical properties to screen for promising c_AFPs. Forty‐nine promising c_AFPs are screened, and their minimum inhibitory concentrations (MICs) against *C. albicans* are determined to be 3.9–125 µg mL^−1^, of which four leading c_AFPs (AFP‐8, −10, −11, and −13) has MICs of <10 µg mL^−1^ against the four tested pathogenic fungi, and AFP‐13 has excellent therapeutic efficacy in the animal model.

## Graphical abstract

Drug resistance remains a serious threat to human health and the planet's ecology. Using deep learning integrated with quantitative structure‒activity relationship, 49 highly active antifungal peptides are *de novo* designed. In vitro and in vivo studies demonstrate these peptides' potential to minimize the risk of resistance development in pathogens, positioning them as promising candidates for treating fungal infections.

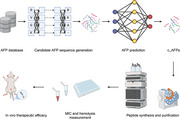

## 1 Introduction

In recent years, microbial infections have continued to cause 1.27 million deaths per year. Unfortunately, despite extensive work on antimicrobial resistance worldwide, drug resistance remains a serious threat to human health and the planet's ecology.^[^ ^1^ ^]^ *Candida albicans*, *Candida tropicalis*, and *Cryptococcus neoformans* are the primary fungal pathogens that infect humans and animals. Only four main classes of antifungal drugs, azoles, polyenes, echinocandins, and fluorinated pyrimidines, are currently available to treat fungal infections,^[^ ^2^ ^]^ and *Candida species* are prone to developing resistance, resulting in difficult‐to‐treat bloodstream infections with a mortality rate of ≈40%.^[^ ^3^ ^]^ Therefore, the development of new antifungal drugs that are less likely to induce resistance in pathogenic fungi is urgent.

Antifungal peptides (AFPs), as emerging antimicrobial drugs, have become the focus of anti‐infective research because of their unique mechanism of action. Unlike traditional antibiotics, AFPs are usually composed of shorter amino acid sequences and have broad‐spectrum antimicrobial activity. They act mainly by disrupting the integrity of the microbial cell wall and cell membrane, biological enzyme activity, deoxyribonucleic acid function, or induction of apoptosis, thus effectively fighting fungal infections.^[^ ^4^ ^]^ More importantly, it is difficult for pathogenic fungi to develop drug resistance. Despite the theoretical potential of AFPs, their development for practical applications still faces many challenges.

Traditional routes for AFP discovery include identifying AFPs following rules of thumb, which usually rely on trial‐and‐error processes and extensive experimental validation. The traditional AFP discovery routes are time‐consuming, laborious, and costly. Notably, with the development of artificial intelligence and computational biology, several high‐throughput computational methods have recently been introduced into the *de novo* design and virtual screening of antimicrobial peptides.^[^ ^5^, ^6^ ^]^ For example, Das et al. used deep learning classifiers and molecular dynamics simulations, among other methods, to screen generated antimicrobial peptides (AMPs).^[^ ^7^ ^]^ Ma et al. combined several natural language processing models to identify candidate AMPs from human gut microbiome data.^[^ ^8^ ^]^ Cheong et al. identified potent anticancer peptides from the *Candida albicans* genome using a machine‐learning approach.^[^ ^9^ ^]^ Huang et al. used a machine learning model to mine hundreds of billions of sequences consisting of 6–9 amino acids from the entire virtual peptide library to identify valid AMPs.^[^ ^10^ ^]^ Yan et al. used Multi‐Branch‐Convolutional Neural Network and Attention for classification and regression prediction of peptides with different functions, respectively.^[^ ^11^, ^12^ ^]^ Cao et al. identified and characterized a variety of AMPs by combining multiple approaches, such as a sequence generation adversarial network, multiple natural language processing models such as Bidirectional Encoder Representations from Transformers (BERT), AlphaFold2, and molecular dynamics simulation.^[^ ^13^ ^]^ Zhang et al. established an effective quantitative structure‒activity relationship (QSAR) model to screen antifungal peptides, which was applied to more than 3 million candidate peptides and identified three prominent peptides.^[^ ^14^ ^]^ Although some research has identified AMPs with high antimicrobial activity against a wide range of test pathogens, there are still many shortcomings in the clinical application of AMPs, such as the low precision of the classification model, the blindness of the antimicrobial spectrum screening of the designed AMPs and the high computational cost of mining the whole virtual peptide library.

The combination of deep learning and QSAR is beneficial in antimicrobial peptide screening.^[^ ^15^ ^]^ In this study, an integrated classification approach of deep learning and QSAR empirical screening (DL‐QSARES) was developed to *de novo* design 49 candidate AFPs (c_AFPs) with high antimicrobial activity against a wide range of tested pathogens, including *C. albicans*, *C. tropicalis*, and *C. parapsilosis*, by computationally analyzing 1237 anti‐*Candida* peptides in databases such as Data Repository of Antimicrobial Peptides (DRAMP) and Antimicrobial Peptide Database 3 (APD3). Notably, these sequences of c_AFPs have not been reported yet. The leading candidates demonstrated excellent therapeutic efficacy in animal models. In conclusion, our work demonstrates the effectiveness of the integrated approach of deep learning and QSAR empirical screening in the field of antifungal peptide *de novo* design and, at the same time, provides new ideas and methods for developing antimicrobial peptide drugs.

## 2 Results

### 2.1 Generation of c_AFPs

**Figure 1:**
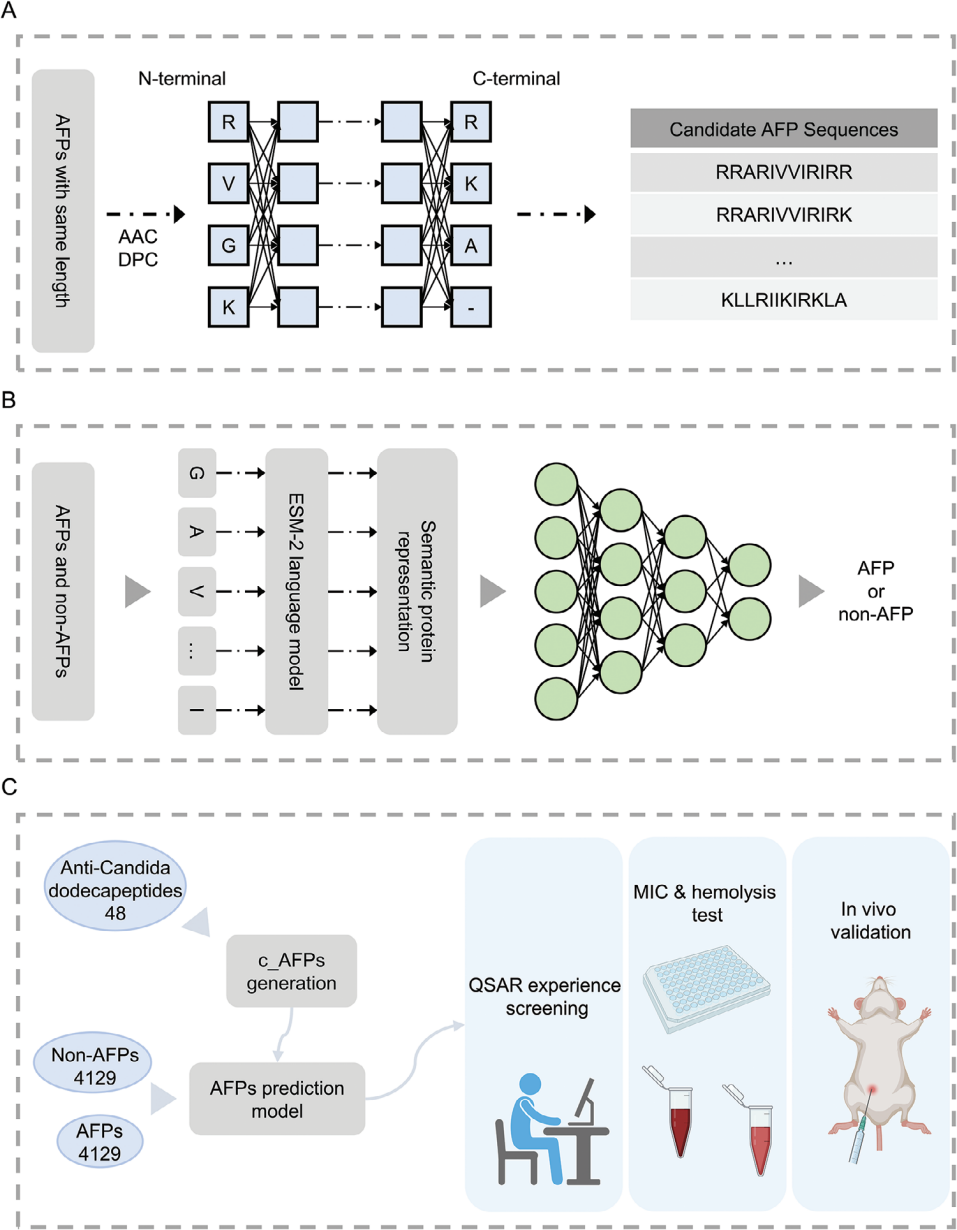
Workflow of the *de novo* design of AFPs using deep learning combined with quantitative structure‒activity relationships. A) New c_AFPs are generated by calculating the amino acid composition (AAC) and dipeptide composition (DPC) parameters at each position of the reported antifungal peptide sequence. B) The Evolutionary Scale Modeling 2‐based antifungal peptide predictor (ESM2‐AFPpred) model consists of two parts, namely, the ESM‐2 and the multilayer perceptron (MLP). ESM‐2 is a protein language model based on BERT pretraining. It receives the contextual semantic information of peptides, processes it into a vector representation, and outputs the predicted probability of the AFP after MLP. C) The novel c_AFPs that successfully passed the ESM2‐AFPpred model and QSAR empirical screening were subjected to In vitro and in vivo experiments to validate their effects.

**Figure 2:**
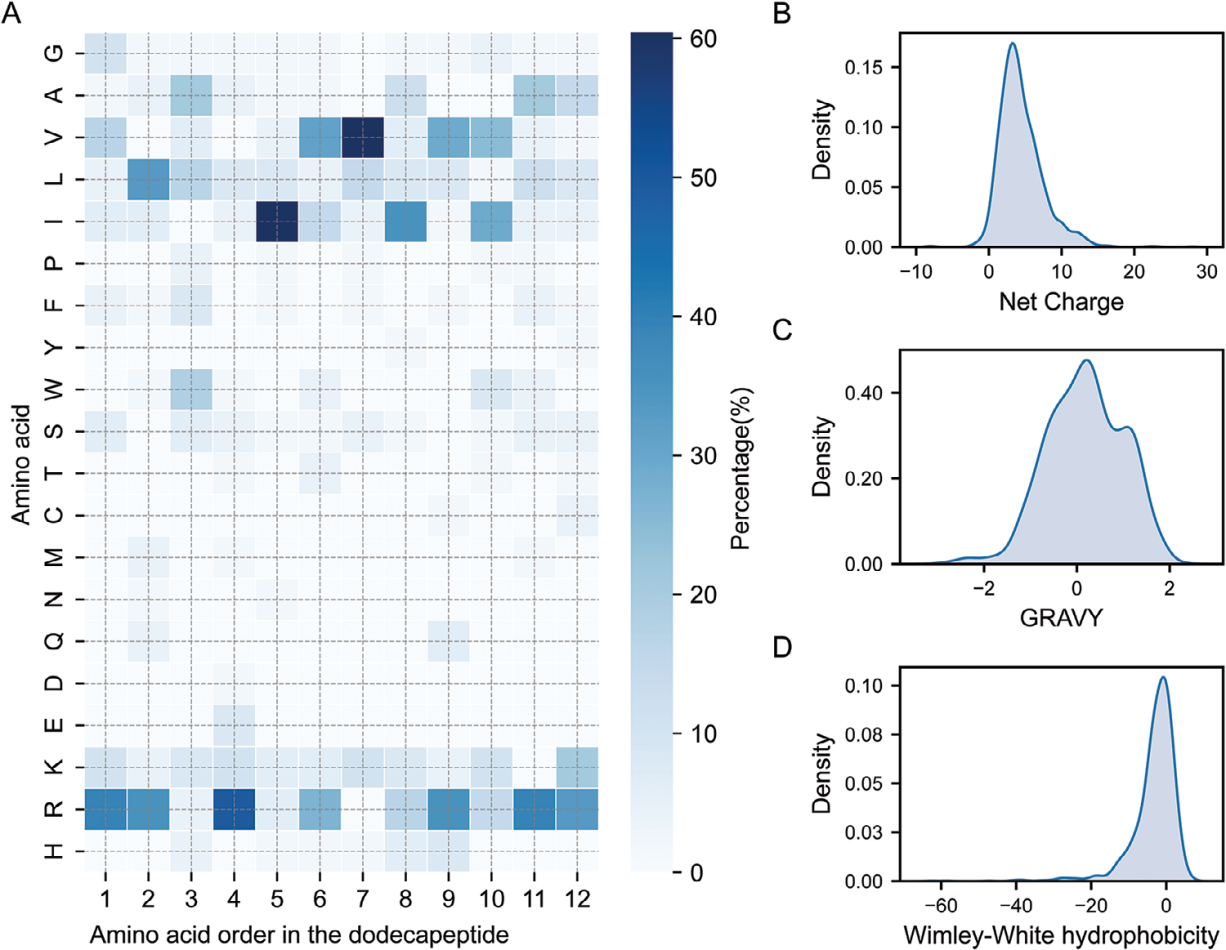
Appearance frequency of amino acids at each position of the anti‐*Candida* dodecapeptide sequence and the distribution of physicochemical properties. A) The frequency of amino acid residues at each position of the 48 anti‐*Candida* dodecapeptide sequences used for the *de novo* design of c_AFP sequences. B) Density plot of the Net Charge data distribution of 1237 anti‐*Candida* peptides. C) Density plot of the GRAVY data distribution of 1237 anti‐*Candida* peptides. D) Density plot of the Wimley‐White hydrophobicity data distribution of 1237 anti‐*Candida* peptides.

By calculating the frequency of occurrence of amino acids and dipeptide compositions at each position of the known anti‐*Candida* dodecapeptides, we regarded the parts that met the set threshold at each position as the dominant amino acids or dominant dipeptide compositions and then arranged and combined these amino acids to generate 7776 candidate peptides, listed on Sheet 1 in Data S1 (Supporting Information). These sequences subsequently underwent a series of virtual screening steps, including screening based on deep learning prediction models, the density distribution of three physicochemical properties, and the Basic Local Alignment Search Tool (BLAST) sequence novelty. The candidate peptides obtained after all the screening steps were subjected to wet experiments to verify their In vitro and in vivo activities. The candidate peptides screened in each step are listed in Sheets 2–4 in Data S1 (Supporting Information). The framework for c_AFP sequence generation is shown in **Figure** **1**A. **Figure** **2** shows the distribution of the dominant amino acids at each position of the 48 anti‐*Candida* dodecapeptides used for c_AFP sequence generation and the density distributions of three physicochemical properties， Net Charge, Grand Average of Hydropathy (GRAVY), and Wimley‐White hydrophobicity, of the anti‐*Candida* peptides in the APD3 and DRAMP databases. The specific dominant amino acid settings and physicochemical property thresholds are shown in Tables S1–S3 (Supporting Information).

### 2.2 Model Training

**Figure 3:**
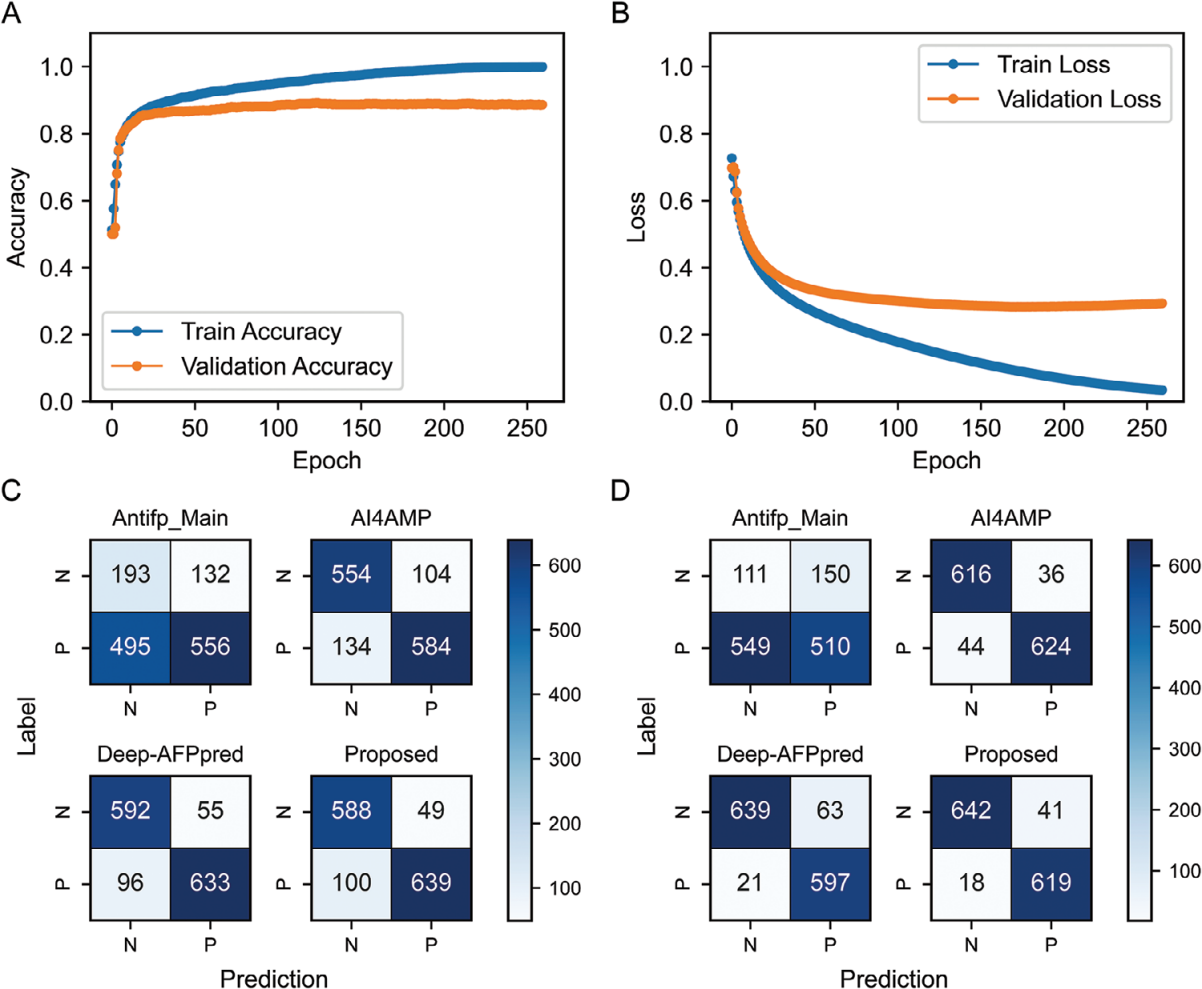
Learning curves of the ESM2‐AFPpred model and confusion matrix plots of the four models. A) Accuracy curves of the ESM2‐AFPpred model. B) Loss curves of the ESM2‐AFPpred model. C) Confusion matrix plots of Antifp_Main (a support vector machine predictor based on compositional features of peptides), AI4AMP (a deep learning predictor based on protein‐encoding method), Deep‐AFPpred (a deep learning algorithm‐based predictor), and ESM2‐AFPpred (an Evolutionary Scale Modeling 2‐based antifungal peptide predictor) confusion matrix plots on the validation set. D) Confusion matrix plots of Antifp_Main, AI4AMP, Deep‐AFPpred, and ESM2‐AFPpred on the independent test set.

The underfitting or overfitting during model training can be effectively judged by comparing the learning curves with the number of epochs. **Figure** **3**A,B shows the model's learning accuracy and loss curves on the training and validation sets. As the number of training rounds increases, the training accuracy continues to increase, and the model's accuracy on the validation set reaches a maximum after 124 epochs and then decreases slightly. The training loss of the model continues to decrease, whereas the validation loss starts to increase slowly after it drops to the lowest point. This indicates that the model reaches its optimal performance after 124 epochs, and the model file with the highest validation accuracy was saved for subsequent screening of c_AFPs. The framework of the model is shown in Figure 1B. The model runtime environment and hyperparameter settings are shown in Table S4 (Supporting Information). The Supplementary text and Figures S1–S3 (Supporting Information) provides more information on the model hyperparameter tuning method.

### 2.3 Comparison with the State‐Of‐The‐Art (SOTA) Model

**Tables** **1**,**2** and Figure 3C,D, show that our proposed model ESM2‐AFPpred (an Evolutionary Scale Modeling 2‐based antifungal peptide predictor) performs well compared with the SOTA model in terms of almost all the performance metrics on the validation set and the independent test set. For the validation set, the second‐best performer is Deep‐AFPpred (a deep learning algorithm‐based predictor),^[^ ^11^ ^]^ which achieves a Recall of 86.05%, which is 0.58% higher than that of our proposed model. However, our model outperforms Deep‐AFPpred in all three other performance metrics. On the independent test set, the second‐best performer is AI4AMP (a deep learning predictor based on protein‐encoding method),^[^ ^12^ ^]^ which achieves a Precision of 94.48%, which is 0.48% higher than that of our proposed model, but our proposed model is the best in terms of the other three performance metrics. The poor performance of Antifp_Main (a support vector machine predictor based on compositional features of peptides) may be due to its use of a limited dataset of only 1585 AFPs from the DRAMP database and does not consider the length of the AFPs used for training,^[^ ^13^ ^]^ whereas the testing data used for other models contain multiple sources of AFPs from different databases, where the peptide length is confined to 5–30 amino acid.

**Table 1:** Performance comparison of the models on the validation set. Deep‐AFPpred is a deep learning algorithm‐based predictor; AI4AMP is a deep learning predictor based on protein‐encoding method; Antifp_Main is a support vector machine predictor based on compositional features of peptides; ESM2‐AFPpred is an Evolutionary Scale Modeling 2‐based antifungal peptide predictor.

	Acc[%]	Pre[%]	Rec[%]	MCC	Algorithm
Deep‐AFPpred^[^ ^16^ ^]^	89.03%	91.50%	**86.05**%	0.7819	1DCNN‐BiLSTM
AI4AMP^[^ ^17^ ^]^	82.70%	84.19%	80.52%	0.6547	CNN‐LSTM
Antifp_Main^[^ ^18^ ^]^	54.43%	59.38%	28.05%	0.1044	SVM
ESM2‐AFPpred	**89.17**%	**92.31**%	85.47%	**0.7856**	BERT‐MLP

**Table 2:** Performance comparison of the models on the independent test set. Deep‐AFPpred is a deep learning algorithm‐based predictor; AI4AMP is a deep learning predictor based on protein‐encoding method; Antifp_Main is a support vector machine predictor based on compositional features of peptides; ESM2‐AFPpred is an Evolutionary Scale Modeling 2‐based antifungal peptide predictor.

	TP	FP	TN	FN	Acc[%]	Pre[%]	Rec[%]	MCC
Deep‐AFPpred^[^ ^16^ ^]^	639	63	597	21	93.64%	91.03%	96.82%	0.8745
AI4AMP^[^ ^17^ ^]^	616	36	624	44	93.94%	**94.48**%	93.33%	0.8789
Antifp_Main^[^ ^18^ ^]^	111	150	510	549	47.05%	42.53%	16.82%	−0.0742
ESM2‐AFPpred	642	41	619	18	**95.53**%	94.00%	**97.27**%	**0.9112**

### 2.4 Screening of c_AFPs

Of the 7776 candidate dodecapeptides generated by our ESM2‐AFPpred model, 3609 were excluded because the model predicted them to be non‐AFPs or predicted them to be AFPs with a probability <0.9. Subsequent screening based on the density distributions of the three physicochemical properties (Net Charge, GRAVY, and Wimley‐White hydrophobicity) revealed 359 c_AFPs that simultaneously met the thresholds for all three. BLAST was used to eliminate sequences with high similarity to known sequences in the nonredundant protein sequence (nr) database to ensure the novelty of the *de novo* designed c_AFPs. Ultimately, 49 c_AFPs were retained. The BLAST results of all 49 c_AFPs are detailed on Sheet 4 of Data S1 (Supporting Information). The detailed c_AFP parameters are shown in Sheets 1–4 of Data S1 (Supporting Information).

### 2.5 Antifungal Activity and Structural Analysis of c_AFPs

The antifungal activity of the final 49 c_AFPs was measured experimentally using *C. albicans* as the primary test pathogen. Notably, all 49 c_AFPs show varying degrees of anticandidal activity, with minimum inhibitory concentrations (MICs) below 125 µg mL^−1^, as detailed in Table S5 (Supporting Information). The four most promising c_AFPs with MICs below 10 µg mL^−1^ are AFP‐8, AFP‐10, AFP‐11, and AFP‐13. Their MICs against *C. tropicalis*, *C. parapsilosis*, and *C. neoformans* were subsequently tested. As shown in **Table** **3**, the MICs of these four c_AFPs were below 10 µg mL^−1^ against all the tested pathogens. Among them, AFP‐10 had the greatest inhibitory effect, with a MIC of 3.51 µg mL^−1^ against *C. albicans*. The four peptides inhibited *C. tropicalis*, with MICs ranging from 2.29–3.62 µg mL^−1^. The best inhibitory agent against *C. parapsilosis* is AFP‐10, with a MIC of 2.61 µg mL^−1^, and the most significant inhibitory agent against *C. neoformans* is AFP‐13, with a strikingly low MIC of 1.89 µg mL^−1^.

**Table 3:** Antifungal and hemolytic activities of c_AFPs.

Peptide	MIC_100_ [µg mL^−1^]				MHC_5_ [µg/mL]^a)^	TI^b)^
	*C. albicans*	*C. tropicalis*	*C. parapsilosis*	*C. neoformans*		
AFP‐8	7.67 ± 0.10	3.62 ± 0.14	2.86 ± 0.41	3.77 ± 0.07	41.81 ± 1.88	5.45
AFP‐10	3.51 ± 0.09	2.32 ± 0.12	2.61 ± 0.33	3.80 ± 0.04	48.11 ± 1.54	13.71
AFP‐11	7.48 ± 0.32	2.35 ± 0.27	5.80 ± 0.88	2.92 ± 0.37	46.51 ± 2.24	6.22
AFP‐13	6.26 ± 0.34	2.29 ± 0.21	9.32 ± 0.28	1.89 ± 0.04	99.50 ± 3.28	15.89

^a)^ MHC_5_, the minimal hemolytic concentration of the peptide that results in 5% human erythrocyte hemolysis;

^b)^ TI indicates the therapeutic index of c_AFPs against *C. albicans*, TI = MHC_5_/MIC_100_.

**Figure 4:**
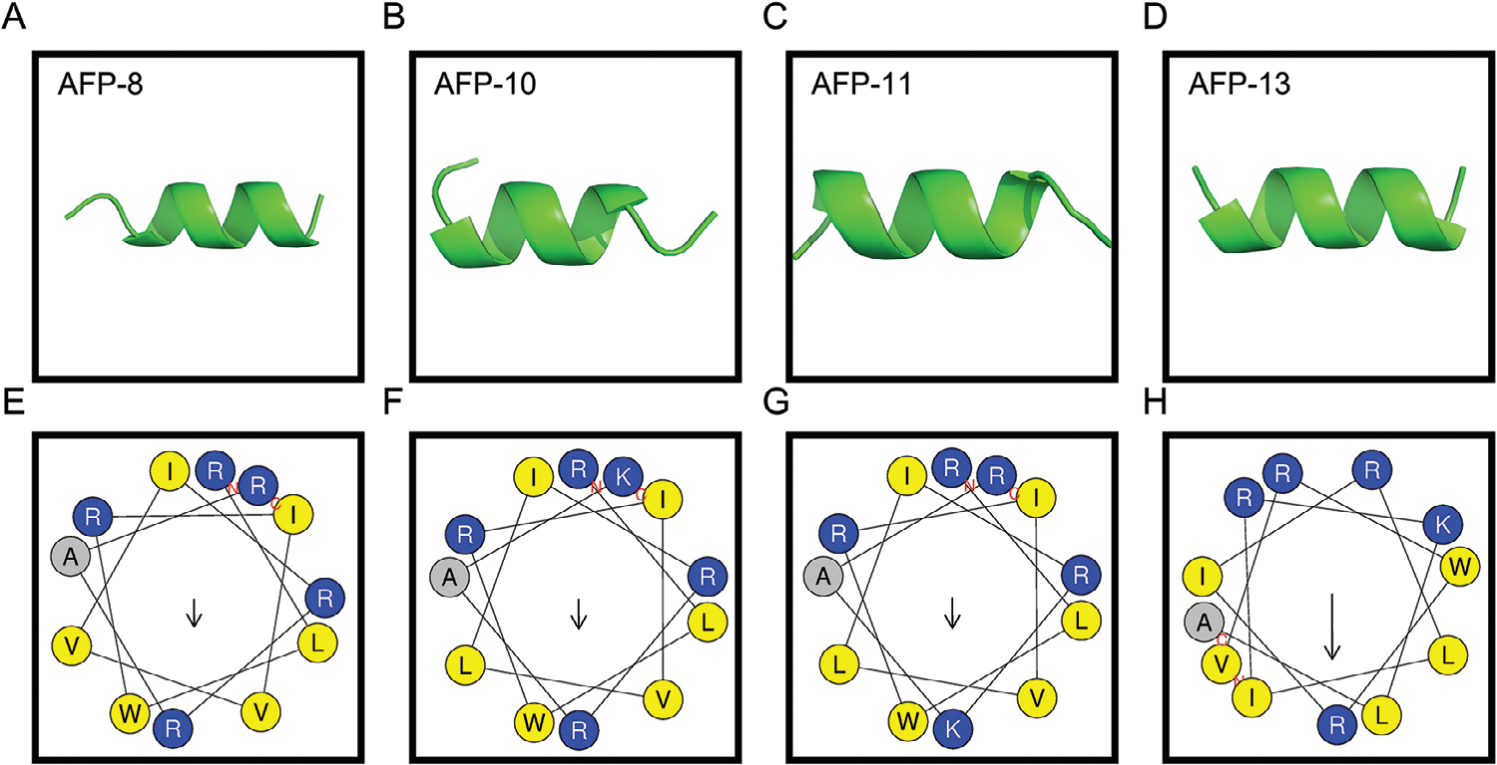
Helical structure diagrams of c_AFPs. A–D) Three‐dimensional structures of the four c_AFPs (AFP‐8, −10, −11, and −13) generated by AlphaFold 3. E–H) Helical wheel diagrams of the four c_AFPs generated by HeliQuest, with positively charged amino acid residues shown in blue and hydrophobic ones in yellow. The arrows indicate helical hydrophobic moments, and the length of the arrow is positively correlated with the hydrophobic moment.

3D structures of the four leading c_AFPs (AFP‐8, −10, −11, and −13) were predicted and generated with AlphaFold 3 (https://golgi.sandbox.google.com/),^[^ ^19^ ^]^ their helical wheel diagrams were predicted and generated by HeliQuest (https://heliquest.ipmc.cnrs.fr/cgi‐bin/ComputParams.py/),^[^ ^20^ ^]^ and their physicochemical properties were predicted by ExPASy (https://web.expasy.org/protparam/).^[^ ^21^ ^]^ The results are shown in **Table** **4** and **Figure** **4**. All four c_AFPs display α‐helical structures and similar physicochemical properties, with both hydrophobic and hydrophilic amino acid residues arranged in two different planes, a structure that favors the activity of these c_AFPs. Notably, the helical wheel diagram of AFP‐13 differs significantly from those of the other three, in which the hydrophilic and hydrophobic amino acids are concentrated on either side of the helical wheel to form a hydrophilic surface and a hydrophobic surface (Figure 4H) and display a significantly stronger hydrophobic moment. This may be one reason why AFP‐13 has the highest antifungal activity among the four peptides.

**Table 4:** BLAST results and physicochemical properties of c_AFPs.

Peptide	Amino acid sequence	Physicochemical properties				BLAST results^a)^		
		Molecular weight	Net charge	GRAVY	Isoelectric point	Max Score	Total Score	Query Cover
AFP‐8	RLWRIVVIRRAR	1593.99	5	−0.033	12.60	34.1	34.1	75%
AFP‐10	RLWRIVLIRRAK	1580.00	5	−0.017	12.48	38.0	38.0	83%
AFP‐11	RLWRIVLIRKAR	1580.00	5	−0.017	12.48	34.6	34.6	75%
AFP‐13	VRWRIRLIRKLA	1580.00	5	−0.017	12.48	33.3	33.3	83%

^a)^ The BLAST results listed are those with the highest Max score values among all the compared sequences.

An alanine scanning experiment was performed to determine the structure‒activity relationship of the amino acid sequence of AFP‐13. As shown in **Table** **5**, the activities of the 11 mutant peptides of AFP‐13 against the four tested pathogenic fungi were lower than those of AFP‐13, which indicated that the sequence structure of AFP‐13 was effective.

**Table 5:** Antifungal activities of Alanine mutants of AFP‐13.

Amino acid sequence	Peptide	MIC_100_ [µg mL^−1^]^a)^			
		*C. albicans*	*C. tropicalis*	*C. parapsilosis*	*C. neoformans*
ARWRIRLIRKLA	AFP‐13[1VA]	15.63	3.91	15.63	3.91
VAWRIRLIRKLA	AFP‐13[2RA]	15.63	3.91	15.63	3.91
VRARIRLIRKLA	AFP‐13[3WA]	31.25	3.91	31.25	7.81
VRWAIRLIRKLA	AFP‐13[4RA]	15.63	3.91	15.63	3.91
VRWRARLIRKLA	AFP‐13[5IA]	62.50	3.91	62.50	3.91
VRWRIALIRKLA	AFP‐13[6RA]	15.63	3.91	31.25	1.95
VRWRIRAIRKLA	AFP‐13[7LA]	125	7.81	31.25	15.63
VRWRIRLARKLA	AFP‐13[8IA]	125	7.81	62.50	7.81
VRWRIRLIAKLA	AFP‐13[9RA]	125	15.63	15.63	7.81
VRWRIRLIRALA	AFP‐13[10KA]	15.63	3.91	31.25	3.91
VRWRIRLIRKAA	AFP‐13[11LA]	62.50	7.81	31.25	7.81

^a)^ The MIC_100_ is the concentration of the drug in ungrown fungus wells measured in 96‐well plates by the microdilution method.

### 2.6 Generalizability of DL‐QSARES

To demonstrate the generalizability of the DL‐QSARES method developed in this paper for peptides of different lengths, a tridecapeptide library was *de novo* designed. The experimental results revealed that all 37 c_AFPs have antifungal activity against the three tested *Candida spp*. (*C. albicans*, *C. tropicalis* and *C. parapsilosis*), four of which have MICs of <10 µg mL^−1^ against *C. albicans*, and almost all of which have MICs of <10 µg mL^−1^ against *C. tropicalis*. The detailed antifungal profiles can be found in Table S6 (Supporting Information).

In addition, we randomly shuffled the sequences of 4 tridecapeptides (AFP‐T3, AFP‐T12, AFP‐T21, and AFP‐T22) with MICs less than 10 µgmL^−1^ to obtain r_AFP‐T3, r_AFP‐T12, r_AFP‐T21, and r_AFP‐T22. The activities of the four reshuffled peptides against the three tested pathogenic fungi (*C. albicans*, *C. tropicalis*, and *C. parapsilosis*) decreased (**Table** **6**), indicating that the DL‐QSARES method is efficient.

**Table 6:** Antifungal activity of r_AFPs with shuffled sequences.

Amino acid sequence	Peptide	MIC_100_ [µg mL^−1^]^a)^		
		*C. albicans*	*C. tropicalis*	*C. parapsilosis*
HGALKFLHLLFKP	r_AFP‐T3	250	31.25	62.50
IRFPLFLKAKGIK	r_AFP‐T12	125	31.25	125
KRLFAKKLVFLPG	r_AFP‐T21	125	31.25	62.50
GAKKLLFPFLLKK	r_AFP‐T22	125	31.25	62.50

^a)^ The MIC_100_ is the concentration of the drug in ungrown fungus wells measured in 96‐well plates by the microdilution method.

### 2.7 Assessment of Resistance Induction and Inhibition of Fluconazole‐Resistant *C. Albicans* by c_AFPs

**Figure 5:**
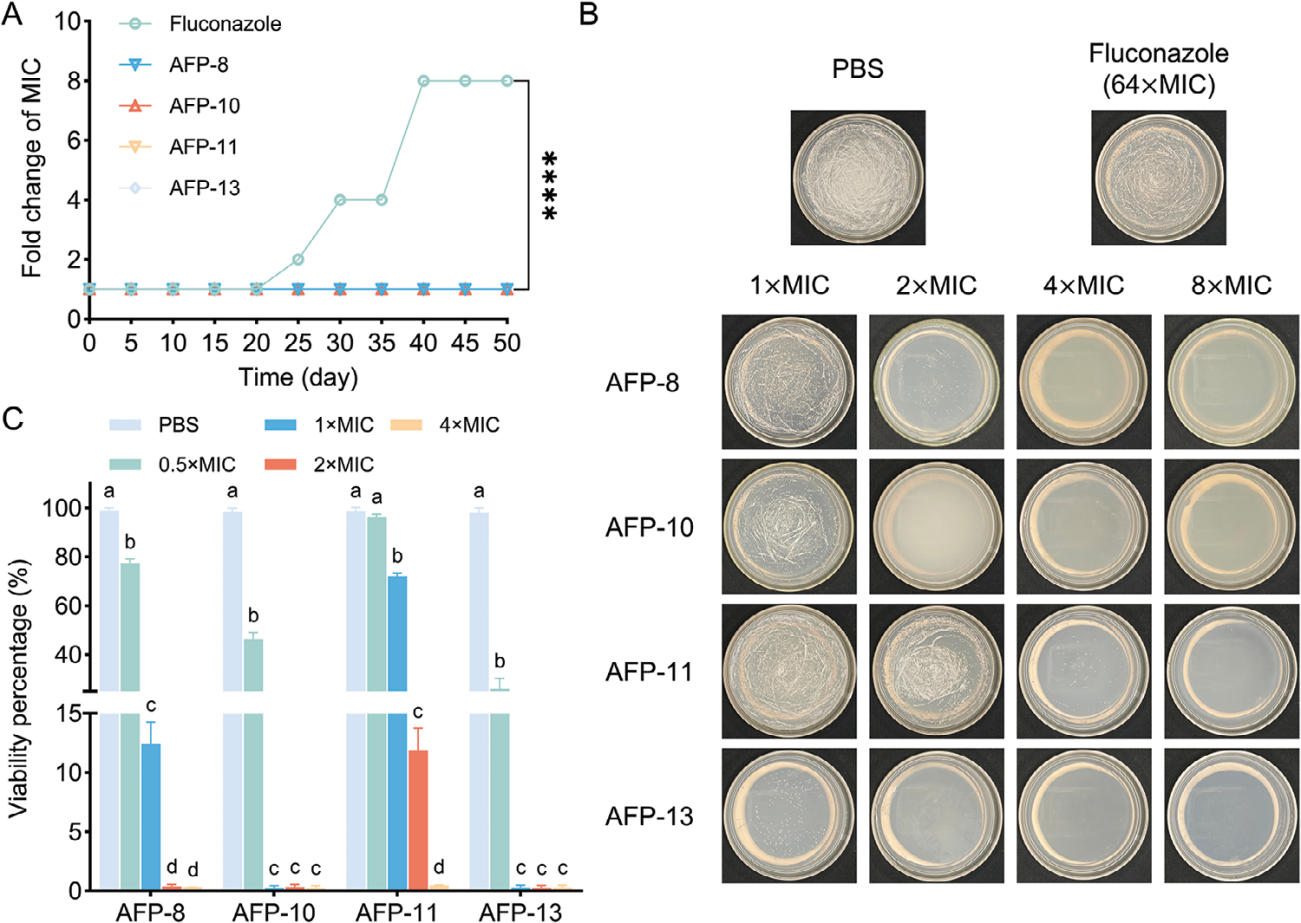
Resistance induction and killing activity against fluconazole‐resistant *C. albicans*. A) Resistance induction of *C. albicans* to c_AFPs. ^****^ *p* < 0.0001. B) Growth of fluconazole‐resistant *C. albicans* after treatment with c_AFPs. C) Inhibition rate of fluconazole‐resistant *C. albicans* by c_AFPs.

Fluconazole resistance is one of the significant challenges in current clinical drug development for treating *Candida* infections. We evaluated the c_AFP resistance propensity of *C. albicans* by a continuous passaging culture method. The results are shown in **Figure** **5**A; the c_AFP groups presented unchanged MICs within 50 generations. In contrast, the MIC of fluconazole (1.9 µg mL^−1^) remained unchanged for the first 20 generations but increased twofold at the 25th generation and eightfold at the 40th generation. These findings suggest that c_AFPs have a lower tendency to induce resistance than fluconazole does. As shown in Figure 5B, compared with fluconazole, which fails to kill fluconazole‐resistant *C. albicans* at even 50 × MIC, the minimum fungicidal concentrations (MFC) are 2 × MIC for both AFP‐10 and AFP‐13, 4 × MIC for AFP‐8 and 8 × MIC for AFP‐11. The inhibitory activity of c_AFPs against fluconazole‐resistant *C. albicans* was further investigated. The results (Figure 5C) revealed that compared with the other two peptides, AFP‐10 and AFP‐13 inhibited the growth of fluconazole‐resistant *C. albicans* by more than 99% at 1 × MIC, demonstrating that they have greater antifungal activity.

### 2.8 In Vitro Hemolysis Evaluation of c_AFPs

**Figure 6:**
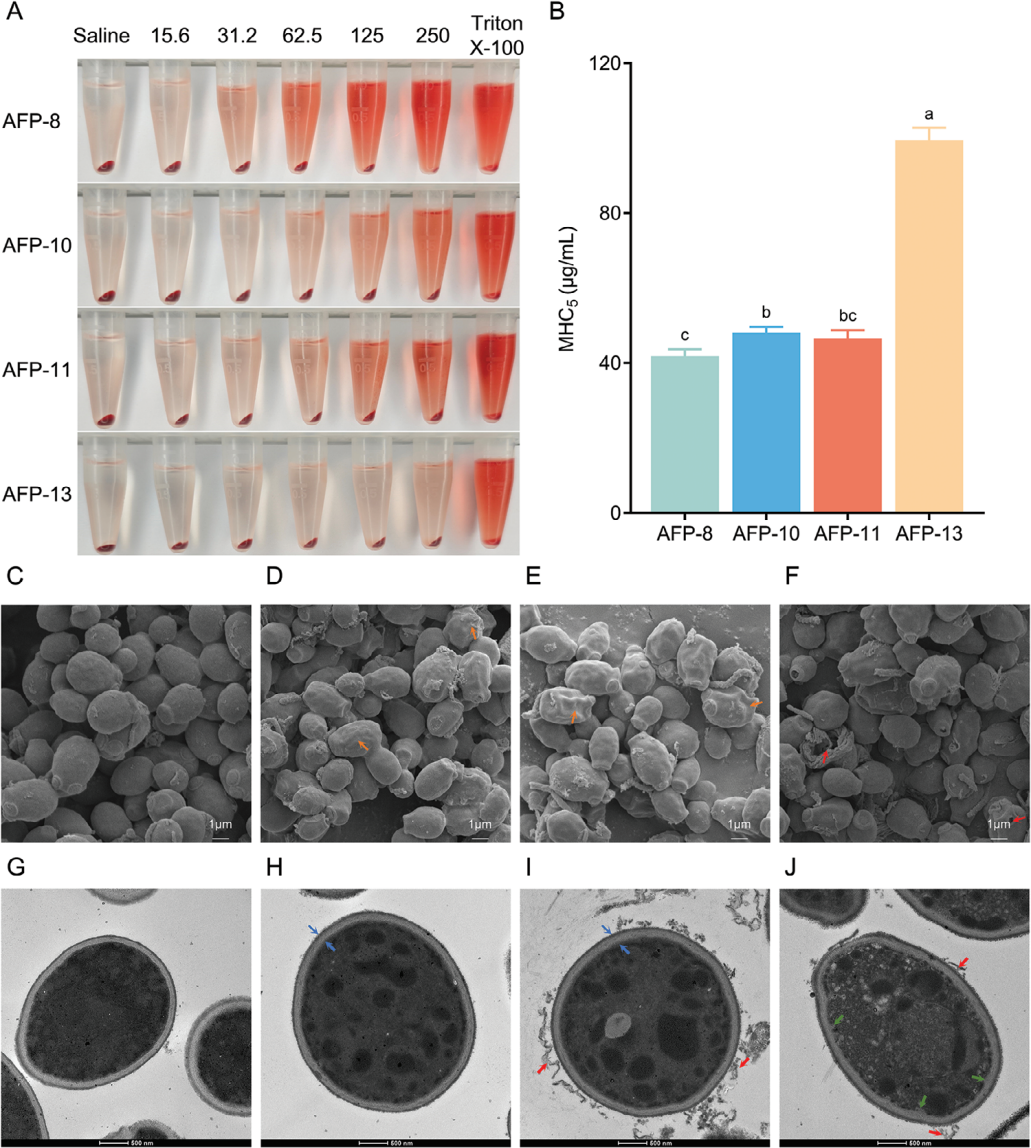
Hemolytic activity of c_AFPs and microstructural changes in *C. albicans* after treatment with AFP‐13. A) Visualization of the hemolytic effect of c_AFPs. B) MHC_5_, the concentration of compounds causing 5% hemolysis of human erythrocytes. C–F) Morphological changes in *C. albicans* after treatment with AFP‐13 for 0, 3, 6, and 9 h under SEM. G–J) Morphological changes in *C. albicans* after treatment with AFP‐13 for 0, 3, 6, and 9 h under TEM.

The effects of c_AFPs on fresh human erythrocytes were evaluated (**Figure** **6**A,B), and the 5% minimum hemolytic concentrations (MHC_5_) of AFP‐8, AFP‐10, and AFP‐11 ranged from 41.81–48.11 µg/mL, with increased hemolysis and decreased biosafety. In contrast, AFP‐13 has high biosafety, with a therapeutic index (TI) of ≈15.89. Safety is positively related to the TI value, which is the ratio of MHC_5_ to MIC_100_ used to assess the biosafety of antifungal peptides. Therefore, AFP‐13 is the most promising candidate for development.

### 2.9 Mechanisms of Action

Morphological and ultrastructural changes in *C. albicans* cells after AFP‐13 treatment were observed by scanning electron microscopy (SEM) and transmission electron microscopy (TEM). The SEM results are shown in Figure 6C–F. As the treatment time increased, the degree of cell shrinkage increased (Figure 6D,E, orange arrows). After 9 h of treatment, the cell wall and membrane ruptured, and the intracellular contents leaked out (Figure 6F, red arrows). The TEM results are shown in Figure 6G–J. Compared with the control group, *C. albicans* cells treated with AFP‐13 for 3 h (Figure 6H) and 6 hours (Figure 6I) showed loose cell walls (blue arrow), unevenly stained cytoplasm, and leakage of intracellular content (red arrow). After 9 h of treatment (Figure 6J), the integrity of the cell membrane was disrupted (green arrow). These results indicate that AFP‐13 exerts its effects by disrupting the cell wall and membrane of fungi in a time‐dependent manner.

### 2.10 In Vivo Therapeutic Efficacy of c_AFPs

**Figure 7:**
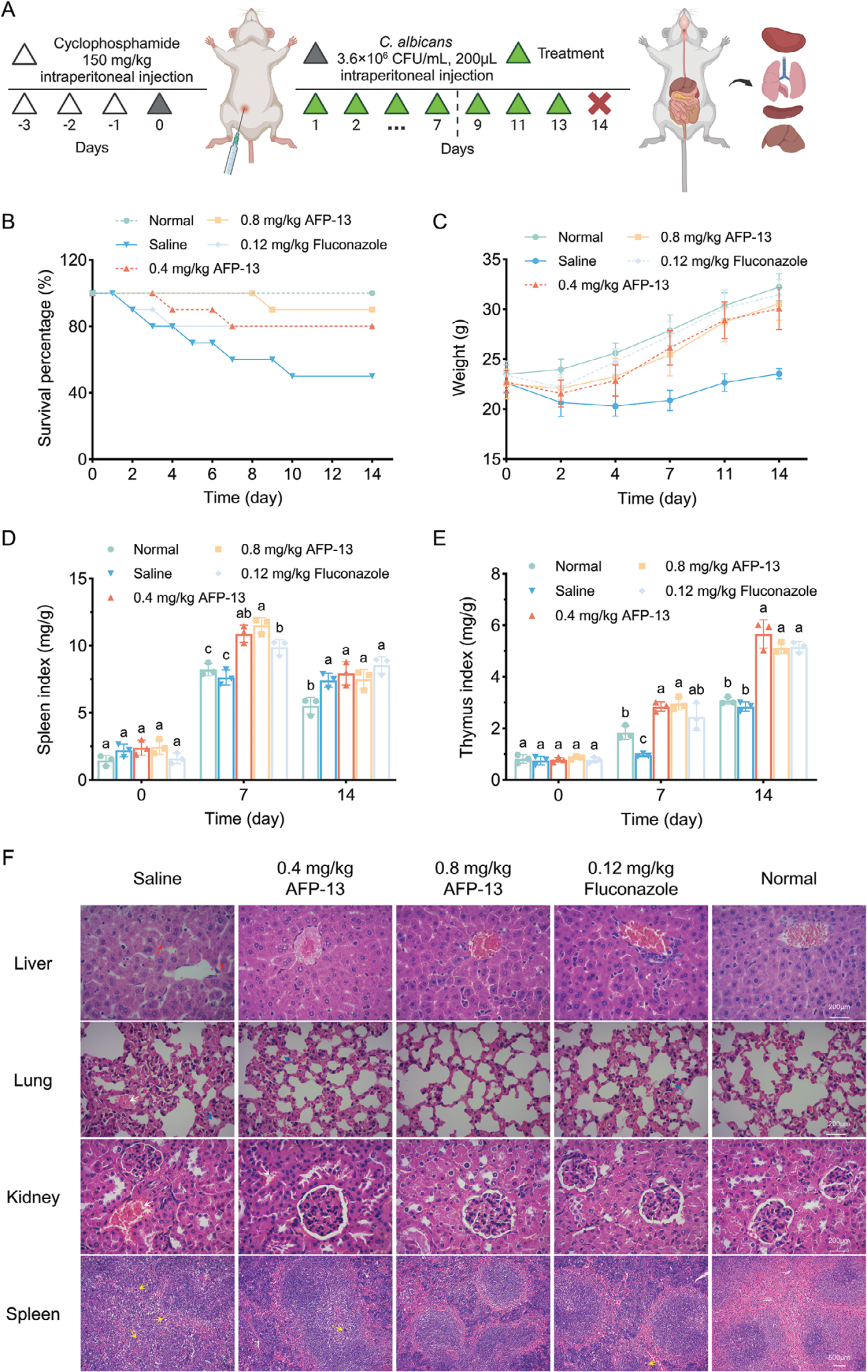
The therapeutic effect of AFP‐13 on a mouse model of *C. albicans* infection. A) Schematic diagram of the construction of the *C. albicans*‐infected mouse model and treatment with AFP‐13. B) Survival rate, *n* = 10. C) Body weight, *n* = 10. D) Splenic index, *n* = 3. E) Thymic index, *n* = 3. F) Tissue sections of the liver, lung, kidney, and spleen after 14 days of treatment. Differently colored arrows indicate nuclear dissolution (red arrow), narrowing of the alveolar lumen (blue arrow), bleeding (white arrow), and macrophages (yellow arrow), respectively.

The in vivo therapeutic efficacy of AFP‐13 was tested in a Kunming mouse model of systemic *C. albicans* infection. **Figure** **7**A shows the construction of the mouse infection model and the drug administration scheme. Figure 7B,C shows that the survival rate of the mice after AFP‐13 treatment was significantly greater than the 50% survival rate of the infected group. The survival rate of the group treated with 0.8 mg kg^−1^ AFP‐13 reached 90%, which was greater than that of the fluconazole group. In addition, the weights of the mice were significantly greater after 14 days of treatment than those of the infected group; the average increased weights of the 0.4 and 0.8 mg kg^−1^ dose groups were 7.58 and 8.00 g, respectively, and that of the fluconazole group was 8.16 g, whereas that of the infected group was 0.76 g.

The immune organ index is an essential indicator of the body's immune function. An increase in the relative weight of immune organs against body weight usually indicates enhanced immune function and increased ability of the immune system to fight pathogenic microorganisms.^[^ ^22^ ^]^ As shown in Figure 7D, AFP‐13 significantly increased the spleen index on Day 7 of treatment. Compared with the splenic index (7.64 mg g^−1^) in the infected group, the values reached 10.89 and 11.51 mg g^−1^ in the 0.4 mg kg^−1^ and the 0.8 mg kg^−1^ AFP‐13 groups, respectively. That of the fluconazole group was 9.89 mg g^−1^. On Days 7 and 14 of treatment, the spleen indices of the AFP and fluconazole groups tended to decrease because of the relatively slower increase in spleen weight and the rapid increase in body weight of the mice. In contrast, the spleen index was relatively stable in the infected group of mice because of the slow increase in body weight. The effects on the thymic index are shown in Figure 7E. On Day 7, the thymic indices of the mice in the 0.4 and 0.8 mg kg^−1^ AFP‐13 groups were 2.85 and 2.96 mg g^−1^ greater than that of the fluconazole group, which was 2.45 mg g^−1^. On Day 14, the thymic indices of the mice in the 0.4 and 0.8 mg kg^−1^ dose groups reached 5.66 and 5.11 mg g^−1^, respectively, which were significantly greater than the 2.85 mg g^−1^ in the infected group and comparable to the 5.16 mg g^−1^ in the fluconazole group. These results indicate that AFP‐13 can significantly stimulate the function of immune organs and thus promote the recovery of infected mice.

Finally, the mouse organ sections on the 14th day of treatment were observed and analyzed. The pathological changes in the internal organs of the mice in each group were observed under a light microscope. The results are shown in Figure 7F. The mice in the infected group presented the most severe tissue lesions. The liver tissue sections exhibited nuclear lysis and the disappearance of typical lobular structures. Lung tissue sections show severe alveolar interstitial lymphocytic infiltration with congestion. In splenic tissue, macrophages are scattered throughout the splenic parenchyma, with a blurred border between the red and white medulla. Hemorrhage was observed in renal tissue sections with glomerular swelling. In contrast, the organ tissues of the mice in the fluconazole and AFP‐13 groups normalized after treatment. Compared with that in the infected group, the hepatic tissue structure in the 0.4 and 0.8 mg kg^−1^ AFP‐13 groups improved after 14 days of treatment. Interstitial lung congestion was reduced, and the alveolar lumen was enlarged. There was no significant hemorrhage in the renal tissue, and the glomerular structure normalized. The germinal center of the spleen was visible. These results indicate that AFP‐13 can effectively reduce the pathological damage to organs caused by systemic *C. albicans* infection.

## 3 Discussion

An antifungal tridecapeptide library was *de novo* designed using the DL‐QSARES method established in this study. Five tridecapeptides (ACP1–ACP5) were the most promising, with notable MICs of 3.9–15.6 µg mL^−1^ against *C. albicans*. These peptides also showed killing effects on *C. albicans* within 8 h at concentrations ranging from 2 to 4 × MIC,^[^ ^23^ ^]^ indicating that the DL‐QSARES method we established is reliable.

The application of artificial intelligence (AI) in the field of drug design is expanding, significantly accelerating the process of new drug discovery and development.^[^ ^24^ ^]^ Traditional drug design usually takes a long time and many resources. AI can efficiently process massive amounts of chemical and biological data to mine potential drug molecules from them through machine learning, deep learning, etc. AI can play an essential role in compound screening, drug target identification, and prediction of drug‐target interactions.^[^ ^25^, ^26^, ^27^, ^28^ ^]^ Through deep learning models, AI can predict the physicochemical properties and biological activities of molecules, optimize the structure of lead compounds, and improve the efficiency of drug development.^[^ ^29^, ^30^ ^]^ In addition, AI has also shown great potential in personalized drug design, assisting in the development of precision drugs for specific patient groups by analyzing patients' genomic data.^[^ ^31^, ^32^ ^]^ Although the application of AI in drug design still faces challenges, such as data quality and model interpretability, with technology advancements and data accumulation, AI is expected to become an essential tool for drug development in the future, further promoting innovation and marketability of new drugs.

The goal of the deep learning‐based *de novo* design framework for AFPs is to screen c_AFPs with specific functions in the massive peptide sequence space. Most AMPs consist of 5–30 amino acids. Taking the virtual search space of octapeptides as an example, there will be more than 20 billion possibilities for random permutations and combinations of 20 natural amino acids, and the search space will grow exponentially with the length of the peptide chain. The screening of c_AFPs with specific functions is challenging, even with the addition of ultrahigh‐precision deep learning prediction models. Compared with Huang et al., who identified effective antimicrobial peptides among hundreds of billions of sequences in the entire virtual peptide library,^[^ ^10^ ^]^ the DL‐QSARES developed in this study narrows the search space of the practical virtual peptide library and identified 49 effective AFPs from the search space consisting of only 7776 sequences, which is less computationally expensive and more efficient. Compared with the identification of candidate AMPs from human gut microbiome data by Ma et al.,^[^ ^8^ ^]^ the method proposed in this study is more selective for the subject pathogens of functional peptides, which enables specific *de novo* design of specified functional peptides with excellent therapeutic effects. Das et al. used deep generative models combined with molecular dynamics simulations to generate and screen 20 candidate antimicrobial peptides within 48 days and ultimately identified two peptides with high activity (7.8–125 µg mL^−1^); our method is more efficient than theirs, and the resulting peptides have better activity.^[^ ^7^ ^]^ Compared with the research of Jin et al., which used molecular docking and molecular dynamics simulations to design functional peptides, our method has obvious advantages in design efficiency. However, in the research of Jin et al., molecular docking and molecular dynamics simulations were used to evaluate the interaction stability and binding affinity between peptides and target proteins, yielding high biological interpretability.^[^ ^33^ ^]^ Wang et al. designed a variety of broad‐spectrum peptides by combining generative models with predictive models. In comparison, our method can generate candidate peptides of specific lengths and functions by controlling the generation algorithm, which is more conducive to the *de novo* design of specific peptides.^[^ ^34^ ^]^

Deep learning models have shown significant advantages in prediction and design tasks. However, the transparency of their internal decision‐making process is insufficient, especially in extracting key sequence features and activity prediction. It is still difficult to clearly explain how the model identifies and utilizes these features. In the future, we will explore the integration of several methods, including deep learning, molecular docking, and molecular dynamics simulations, to achieve efficient *de novo* design of highly active antifungal peptides. Moreover, we strive to improve the transparency of the model's decision‐making process and analyze the specific mechanism of the model in key sequence feature extraction and activity prediction through feature visualization and causal analysis.

## 4 Conclusion

We successfully developed a *de novo* design method for c_AFPs called DL‐QSARES. This pipeline generates sequences through the compositions of dominant amino acids and dipeptides, followed by sequence screening using a combination of deep learning and QSAR empirical screening. Using the DL‐QSARES approach, we *de novo* designed 49 c_AFPs, and remarkably, all of them exhibited high antifungal activity (positive rate = 100%) against a wide range of tested pathogens (*C. albicans*, *C. tropicalis*, and *C. parapsilosis*). The AFP prediction model we developed, ESM2‐AFPpred, outperformed SOTA models such as Deep‐AFPpred, AI4AMP, and Antifp_Main in terms of comprehensive performance, which was crucial to our success. In addition, we evaluated the generalizability of the developed DL‐QSARES method using tridecapeptides, and the results demonstrated that our method was effective. Among the 49 promising c_AFPs selected in the final screening, four of the major candidates (AFP‐8, ‐10, ‐11, and ‐13) presented MICs of <10 µg mL^−1^ against the four tested pathogenic fungi. Notably, AFP‐13 demonstrated excellent therapeutic efficacy in a *Candida albicans* infection mouse model. The sequences of these c_AFPs have not been reported, indicating their novelty and potential application. Our study not only provides innovative ideas and methods for the development of antifungal peptides but also lays the foundation for future research in related fields and promotes the development of antifungal drugs.

## 5 Experimental Section

### Dataset

This study's training and validation sets were taken from Deep‐AFPpred.^[^ ^16^ ^]^ In the Deep‐AFPpred dataset, positive samples were collected from the CAMP,^[^ ^35^ ^]^ DRAMP,^[^ ^36^ ^]^ and StarPep^[^ ^37^ ^]^ databases for AFP sequences of length ∈ [5,30], and negative samples were randomly generated from the Swiss‐Prot database. Positive samples for the independent test set were screened from the APD3 database^[^ ^38^ ^]^ for AFP sequences of length ∈ [5,30] that were not duplicated from the training and validation sets. Negative samples were composed of manually reviewed and annotated proteins from the UniPort database that did not contain any of the following keywords: Activator [KW‐0010], Antibiotic [KW‐0044], Antioxidant [KW‐0049], Bacteriolytic enzyme [KW‐0081], Fungicide [KW‐0295], Hormone [KW‐0372], Hypotensive agent [KW‐0382], Lantibiotic [KW‐0425], Antimicrobial [KW‐0929], Antiviral protein [KW‐0930], Antiviral defense [KW‐0051], Repressor [KW‐0678], Protease inhibitor [KW‐0646], Defensin [KW‐0211], Insecticide resistance [KW‐0978], Chemotaxis [KW‐0145], Antifungal, Effector, Wound healing, Spermicidal, Antibacterial Spermicidal, Antibacterial, Antimalarial, Antiparastal, Anticancer, Anti‐HIV. Notably, unlike Deep‐AFPpred, this study repartitioned the training and validation sets at an 8:2 ratio. The former was used to train the model for the classification task of the AFP, and the latter was used to tune the model's hyperparameters. The positive and negative samples in the training and validation sets in this study have the same distribution, and the dataset details are shown in Table S7 and Figure S4 (Supporting Information).

### Antifungal Peptide De Novo Design

The anti‐*Candida* dodecapeptides in APD3 and DRAMP (48 in total) were analyzed by AAC and DPC calculations to obtain the dominant amino acid and dominant dipeptide compositions at each position. AAC and DPC are the basis for determining whether the amino acids present in the fixed positions of existing dodecapeptides are dominant amino acids:

(1) $$\mathrm{AAC}\;=\frac{A\left(x\right)}{\sum_{x=1}^{20}A\left(x\right)}$$

(2) $$\mathrm{DPC}\;=\frac{D\left(x\right)}{\sum_{x=1}^{400}D\left(x\right)}$$

Among them, *A*(*x*) represents all amino acids appearing at each position, and *D*(*x*) represents all dipeptide compositions. In order to reduce the calculation demands and obtain peptides with the highest theoretically predicted antifungal activity, the number of dominant amino acids at each position is controlled to be 1–4. Therefore, the threshold value of the dominant amino acid is set to 10%, and the threshold value of the dominant dipeptide composition is set to 30%.^[^ ^39^ ^]^ Amino acids that satisfy the set threshold were used as the dominant amino acid at each position of c_AFPs, and c_AFP sequences were generated according to the following rules:

(3) $$A1\times A2\times \text{…}\times An=\left\{\left(x1,x2,\text{…},xn\right)|xi\in Ai,i=1,2\text{…},n\right\}$$

where *An* represents the set of dominant amino acids at the *n*th position of the c_AFPs to be generated. The number of generated c_AFPs is determined by the number of dominant amino acids at each position.

Notably, to ensure that the predicted antifungal activity against *Candida spp*. of the c_AFPs obtained from the *de novo* design was greater, the density distributions of three physicochemical properties were analyzed, namely, Net Charge,^[^ ^40^ ^]^ GRAVY,^[^ ^41^ ^]^ and Wimley‐White hydrophobicity,^[^ ^42^ ^]^ for all lengths of anti‐*Candida* peptides from ADP3 and DRAMP. These analyses were then used as a reference for screening c_AFPs.

BLAST (https://blast.ncbi.nlm.nih.gov/Blast.cgi) from the National Center for Biotechnology Information for proteins is a commonly used bioinformatics tool for comparing the similarity of protein sequences. BLAST identifies sequences that are highly similar to the designed sequence by aligning them with known protein sequences in the database.^[^ ^43^ ^]^ Therefore, BLAST helps to screen the designed peptides with novel sequences. Here, BLAST was used to compare the sequences of *de novo* designed c_AFPs with those in the nr database.

### AFP Classification Model

The architecture of the classifier consists of two modules: feature extraction and classification. In the feature extraction module, inspired by the successful application of the transformer‐based pre‐trained model in various protein sequence representation tasks, the pre‐trained protein model ESM‐2 was chosen as a feature extractor for contextual semantic information of peptides.^[^ ^13^, ^44^, ^45^, ^46^ ^]^ ESM‐2 is a protein model trained on a masked language modeling target, and features learned using millions of sequences in the Uniref50 dataset are implemented for classifying AFPs. Then, a MLP was used to obtain the feature representations generated by ESM‐2 and obtain classification results for the AFPs. ESM‐2 automatically extracts high‐dimensional features from peptide sequences that include contextual information about the amino acid sequence and potential functional properties. Additional filtering on these features was not performed but directly used the features extracted by ESM‐2 as input to the MLP for training. The MLP uses contextual semantic features extracted by ESM‐2 as the basis for antifungal peptide classification. The combination of the above two algorithms enables the prediction of antifungal activity for novel sequences.

In this study, Accuracy (Acc), Precision (Pre), Recall (Rec), and Matthews correlation coefficient (MCC) were used as model evaluation metrics, which were calculated as follows:

(4) $$\mathrm{Acc}=\frac{\mathrm{TP}+\mathrm{TN}}{\mathrm{TP}+\mathrm{TN}+\mathrm{FP}+\mathrm{FN}}$$

(5) $$\mathrm{Pre}=\frac{\mathrm{TP}}{\mathrm{TP}+\mathrm{FP}}$$

(6) $$\mathrm{Rec}=\frac{\mathrm{TP}}{\mathrm{TP}+\mathrm{FN}}$$

(7) $$\mathrm{MCC}=\frac{\left(\mathrm{TP}\times \mathrm{TN}\right)-\left(\mathrm{FP}\times \mathrm{FN}\right)}{\sqrt{\left(\mathrm{TP}+\mathrm{FP}\right)\left(\mathrm{TP}+\mathrm{FN}\right)\left(\mathrm{TN}+\mathrm{FP}\right)\left(\mathrm{TN}+\mathrm{FN}\right)}}$$

TP, TN, FP, and FN represent the numbers of true positives, true negatives, false positives, and false negatives, respectively.

### Peptide Synthesis and Purification

The final screened c_AFPs were synthesized by a solid‐phase peptide synthesis method using an amino resin as a carrier and amino acids with protecting groups as raw materials.^[^ ^47^ ^]^ The Rinkamide‐AM resin was first shaking swelled with Dichloromethane (DCM) in a synthesis tube overnight and then incubated with 20% piperidine in N, N‐dimethylformamide (DMF) for 0.5 h to remove the protecting group Fmoc from the resin. The resin was then cross‐cleaned using DCM and DMF three times. Amino acids were sequentially coupled to the resin using DMF as solvent and 1‐Hydroxybenzotriazole /N, N′‐Diisopropylcarbodiimide as coupling agent. After each splicing of amino acids, the Fmoc protecting group was removed with 20% piperidine in DMF, and the process was repeated until all amino acids were coupled and Fmoc was completely spliced. At last, the polypeptides were cleaved from the resin using 1,3‐dimethoxy benzene/triisopropylsilane/methyl phenyl sulfide/phenol/water/trifluoroacetic acid (1:1:2:3:1:35). The peptides were finally precipitated by ice ether to obtain the crude c_AFPs in powder form. The c_AFPs crudes were purified by semi‐preparative high‐performance liquid chromatography (GX‐271, GILSON, USA) on a CAPCELL PAK C18 column (9.4 × 250 mm, 5 µm) using ultrapure water and ethyl ether as mobile phases, with a detection wavelength of 214 nm and a sub‐wavelength of 254 nm.^[^ ^48^ ^]^

### Minimal Inhibitory Concentration (MIC) Measurement

The microdilution method was used to determine the MIC of c_AFPs.^[^ ^49^ ^]^ In 96‐well plates, the candidate peptides were serially diluted in two folds with 100 µL phosphate‐buffered saline (PBS, 20 mm, pH 6.0 for fungi) at the 0–1000 µg mL^−1^ concentration range in each well line. Subsequently, an equal volume of the tested fungal suspension in the logarithmic growth phase(1 × 10^6^ colony forming units mL^−1^, CFU mL^−1^) was added and incubated for 20 h at 30 °C. Sabouraud's Dextrose (SD) broth was set as a blank control, and the fungi suspension without peptides was set as a negative control. The Methylthialazole Tetrazolium staining method was used to calculate the MIC of the screened c_AFPs. Each experiment was replicated three times.

### Hemolysis Assay

A 2% fresh human red blood cells (HRBCs) suspension was used in the hemolysis assay of c_AFPs.^[^ ^48^, ^50^ ^]^ The c_AFPs solution was prepared in saline with a concentration range of 0–0.5 mg mL^−1^. Then, 2% HRBCs suspension was incubated with different concentrations of c_AFPs in a 96‐well plate (37 °C, 30 min). Finally, the amount of hemoglobin released after erythrocyte lysis was measured at 540 nm to calculate the minimum hemolytic concentration of the peptide that results in 5% human erythrocyte hemolysis (MHC_5_). Saline was used as a negative control, and Triton X‐100 (1%) was used as a positive control. Each experiment was replicated three times.

### Assessment of Resistance Induction and Inhibition of Fluconazole‐Resistant *C. albicans* by c_AFPs

The resistance tendency of c_AFPs developed in *C. albicans* was assessed utilizing a continuous passaging culture.^[^ ^23^, ^51^ ^]^ 20 µL of *C. albicans* suspension was added to 2 mL of SD broth containing 0.5 × MIC c_AFPs/antibiotic and incubated for 24 h (28 °C, 180 rpm). Subsequently, 20 µL of culture was transferred to a fresh SD broth with the same concentration of c_AFPs/antibiotic. The concentrations of c_AFPs/antibiotic in the SD broth were doubled every 10 generations, and the MICs of the c_AFPs/antibiotic were detected every 5 generations. Each 24‐hour incubation was considered one generation. PBS (pH 6.0) was used as a blank control, and fluconazole was used as an antibiotic control. Each experiment was performed three times.

Fluconazole‐resistant *C. albicans* was prepared following the established methods.^[^ ^23^, ^51^, ^52^ ^]^ *C. albicans* were cultivated in an SD broth medium containing fluconazole (100 × MIC) for 24 h (28 °C, 180 rpm), and the surviving cells were fluconazole‐resistant *C. albicans*. 1 × 10^6^ CFU mL^−1^ of the fluconazole‐resistant *C. albicans* were treated with c_AFPs (1.9–500 µg mL^−1^) in 96‐well plates (28 °C, 12 h), and the survival rate of the cells at different multiplicities of MIC was determined by the absorbance at 540 nm. Meanwhile, 20 µL of the culture at different multiplicities of MIC was coated on SD agar medium to record the grown colonies (37 °C, 16 h), and the minimum fungicidal concentration, which was the lowest concentration for killing fluconazole‐resistant *C. albicans*, was calculated.

### Mechanism of Action

The morphology and ultrastructure of *C. albicans* cells post‐treatment of c_AFPs were investigated using a previous method.^[^ ^53^ ^]^ *C. albicans* suspension (1 × 10^6^ CFU mL^−1^) was centrifuged and washed with PBS buffer to remove the supernatant. The precipitated cells were incubated with c_AFPs solution (1 × MIC) in SD broth at 28 °C for 3, 6, and 9 h. At the end of the incubation, the supernatant was removed by centrifugation (4 °C, 3000 rpm, 5 min), and the precipitate was subsequently performed SEM and TEM.

For SEM, the cell precipitate was dehydrated in serious concentrations of ethanol (30%, 50%, 70%, 90%, and 100%), ethanol and tert‐butanol mixture (1:1), and pure tert‐butanol. Finally, the samples were freeze‐dried and observed under SEM (Quanta FEG 250, FEI, USA). PBS (0.02 M, pH 7.2) was used as a control.

For TEM, the cell precipitate was successively freeze‐dried, embedded in resin, and sectioned. Subsequently, they were stained using lead and uranium and dried overnight at room temperature before being observed using TEM (Talos L120C, Thermofisher, USA). PBS (0.02 M, pH 7.2) was used as a control.

### In Vivo Therapeutic Efficacy of c_AFPs

The systemic *C. albicans* infection mouse model was established following the reported reference.^[^ ^22^ ^]^ Cyclophosphamide (150 mg kg^−1^ d^−1^) was first injected intraperitoneally for 3 days to disrupt the immunity of female Kunming mice (Experimental Animal Center of Zhengzhou University, SCXK Yu 2021–0009). Five groups were set for in vivo therapeutic efficacy detection of AFP‐13: a negative control group, injected intraperitoneally with 200 µL of 0.9% NaCl, *n* = 30; a fluconazole group, injected intraperitoneally with 0.12 mg kg^−1^ of fluconazole, 200 µL, *n* = 30; and 0.4 mg kg^−1^ dose of AFP‐13 group, injected intraperitoneally with 0.4 mg kg^−1^ of AFP‐13, 200 µL, *n* = 30; 0.8 mg kg^−1^ dose of AFP‐13 group, injected intraperitoneally with 0.8 mg kg^−1^ of AFP‐13, 200 µL, *n* = 30; and a normal mice group, which the health mice were intraperitoneally injected with 0.9% NaCl, 200 µL, *n* = 30. The injections were given once every 24 h during the first week and once every 48 h during the second week. Survival rates and body weights of the mice in each group were recorded during treatment. At 0, 7, and 14 d of treatment, duplicate samples (*n* = 3) of the spleen and thymus were collected from each group to determine the immune organ index.^[^ ^54^ ^]^ The ratio of organ weight to body weight was adopted to describe the immune organ index. At 14 d of treatment, liver, lung, kidney, and spleen tissues were collected from each group of mice and immersed in 4% paraformaldehyde fixative. Subsequently, the tissue samples were embedded in paraffin, followed by sectioned and stained with hematoxylin‐eosin (H&E). Finally, optical microscopy was used to observe the pathological changes in the organ tissues of the mice.

All animal experiments were performed according to the protocols approved by the Institutional Animal Care and Use Committee of Zhengzhou University (Approval No: ZZUIRB 2023–007).

### Statistical Analysis

In this study, Origin 2022 and GraphPad Prism 10.1.2 were used for data processing. The results are presented as the mean ± standard deviation of three independent experiments and evaluated for statistical significance using a one‐way analysis of variance. Intergroup differences are defined as statistically significant when *p* < 0.05 and are represented by different letters. When *p* ≥ 0.05, there is no significant difference between the groups, represented by the same letter.

## Conflict of Interest

The authors declare no conflict of interest.

## Author Contributions

K.Y. and R.L. contributed equally to this work. R.L., D.X., W.X., and L.H. performed conceptualization; K.Y., S.Z., Y.S., and M.J. performed methodology; K.Y., S.Z., and Y.S. performed investigation; K.Y. and R.L. performed visualization; R.L., D.X., and W.X. performed supervision; K.Y. and R.L. write‒original draft; R.L. wrote and reviewed the original draft and edited the manuscript.

## Supporting information



Supporting Information

Supporting Data

## Data Availability

The data that support the findings of this study are openly available in GitHub at https://github.com/DongYin521/AFP_DL‐QSARES.
