# Supplementary material for: Deep Learning Combined with Quantitative Structure‒Activity Relationship Accelerates De Novo Design of Antifungal Peptides
Source: Adv Sci (Weinh). 2025 Feb 8;12(13):2412488. doi: 10.1002/advs.202412488 (PMC11967820; doi:10.1002/advs.202412488)
Supplement: Supplementary file 1 — Supporting Information [file ADVS-12-2412488-s002.docx]

Supporting Information

Deep learning combined with quantitative structure‒activity relationship accelerates *de novo* design of antifungal peptides

Kedong Yin, Ruifang Li*, Shaojie Zhang, Yiqing Sun, Liang Huang, Mengwan Jiang, Degang Xu* and Wen Xu*

**Supplementary Text**

*Tuning of hyperparameters for the ESM2-AFPpred model*: To get the best antifungal peptide prediction, we tuned the hyperparameters of the prediction model to make the prediction model achieve the best performance:

1 An esm2_t6_8M_UR50D model with smaller parameters was used as the pre-training model, and the batch size was set to 128. As shown in Figure S1, the model performed best when the learning rate was set to 0.00001.

2 With esm2_t6_8M_UR50D as the pre-training model, we set the learning rate to 0.00001. As shown in Figure S2, the model performs best when the batch size is 256.

3 We applied a learning rate of 0.00001 and a batch size of 256. As shown in Figure S3, the model performed best when esm2_t30_150M_UR50D was used as the pretrained model.


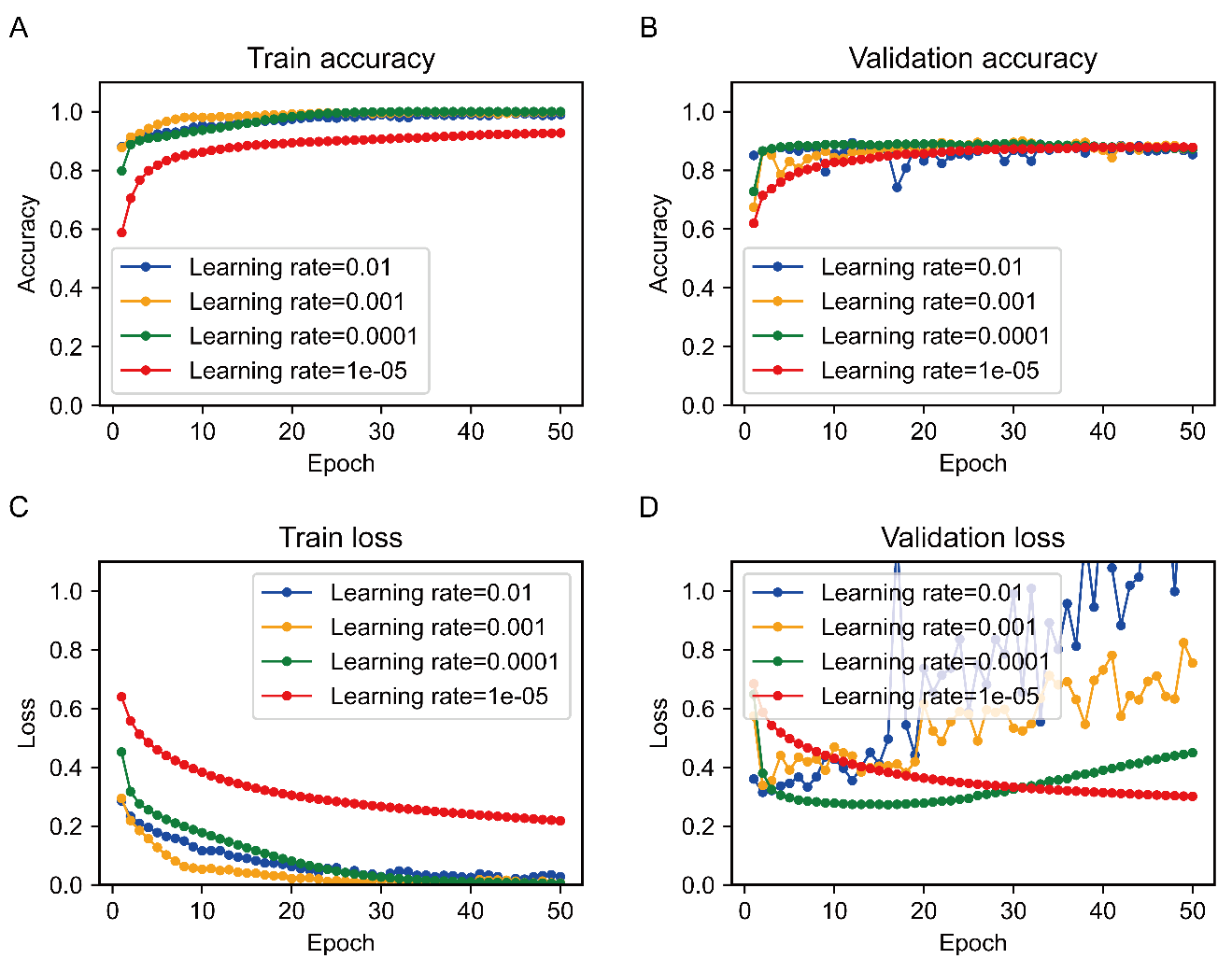


**Figure S1 Tuning the learning rate.**


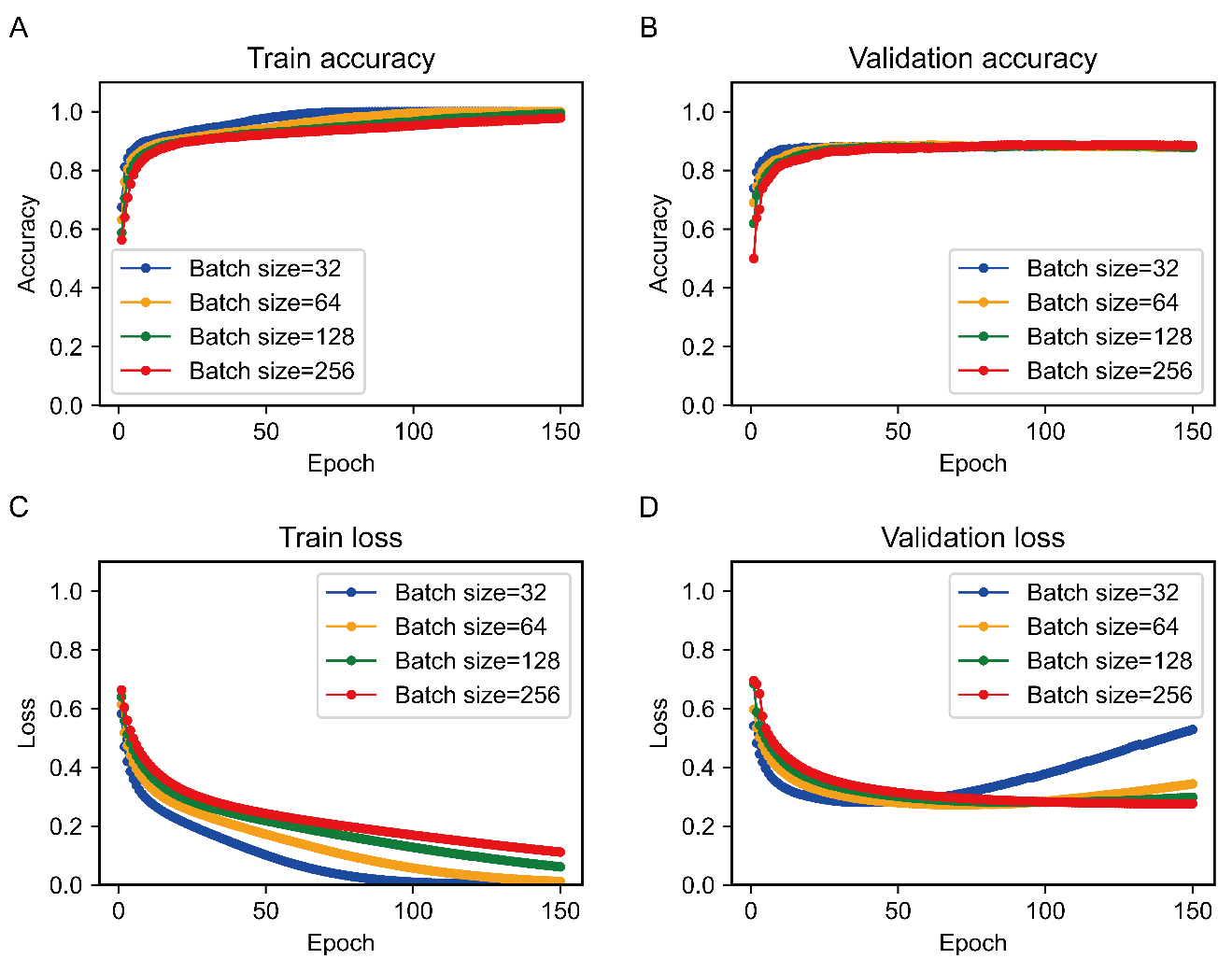


**Figure S2 Tuning the batch size.**


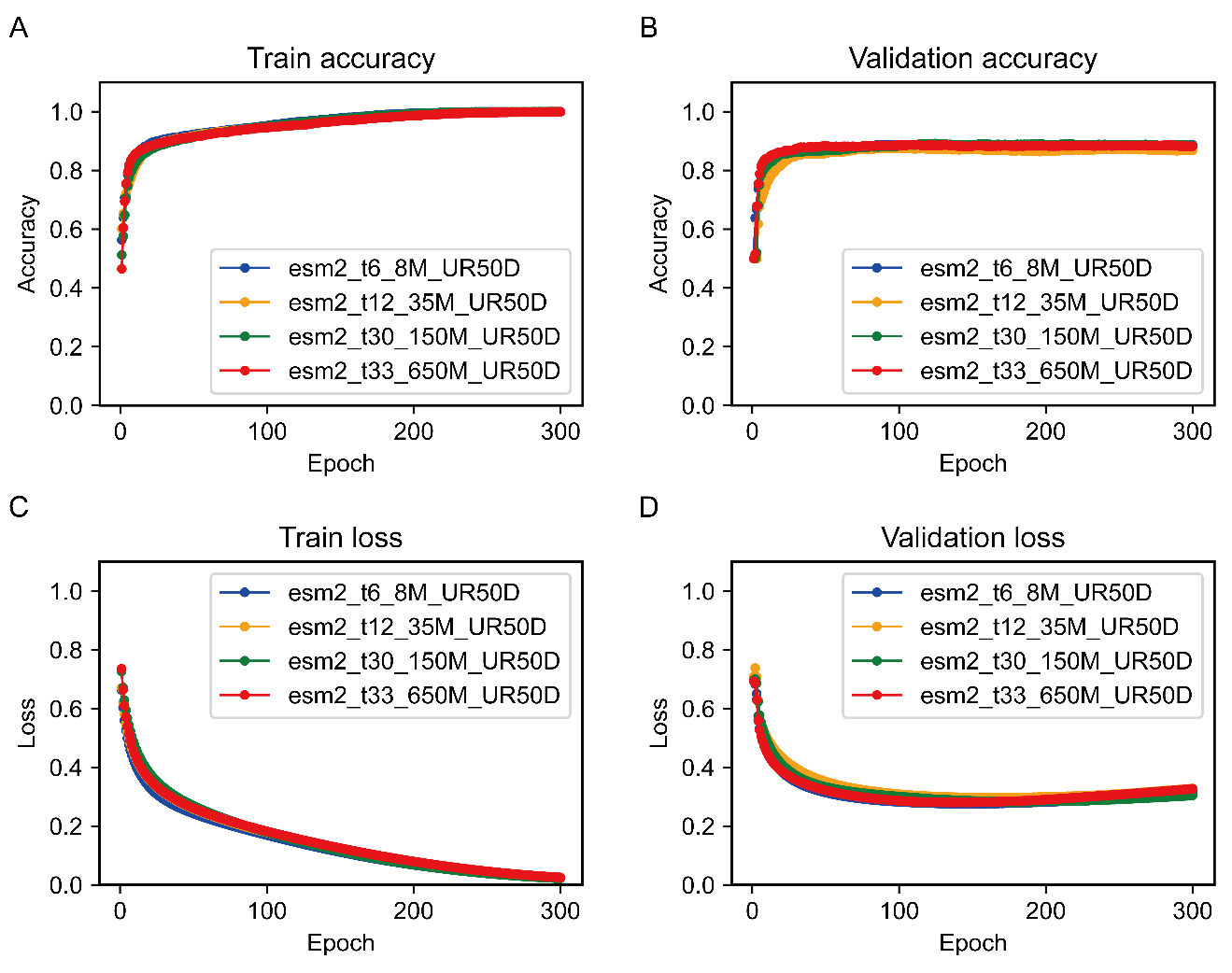


**Figure S3 Tuning the ESM-2 model.**


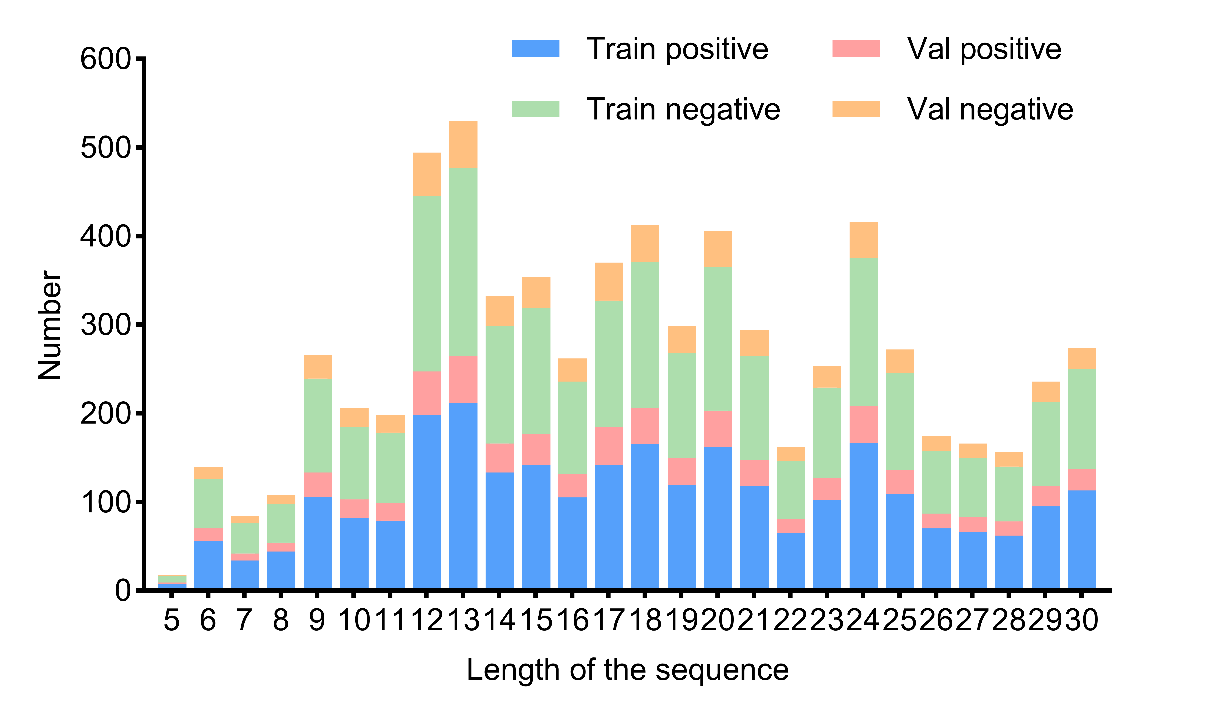


**Figure S4 Sequence length distribution for training and validation sets.**

**Table S1** Dominant amino acid composition of the dodecapeptide sequences.

| Position | Dominant amino acids | Dominant amino acids total quantity |
| --- | --- | --- |
| 1 | R, V, G, K | 4 |
| 2 | R, L | 2 |
| 3 | A, W, L | 3 |
| 4 | R | 1 |
| 5 | I | 1 |
| 6 | V, R, I | 3 |
| 7 | V, L, K | 3 |
| 8 | I | 1 |
| 9 | R | 1 |
| 10 | I, V, R, K | 4 |
| 11 | R, A, L | 3 |
| 12 | R, K, A | 3 |

**Table S2** Dominant dipeptide composition of the dodecapeptide sequences.

| Position | Dominant Dipeptide composition | Frequency (%) |
| --- | --- | --- |
| 4&5 | R (Arg) and I (Ile) | 45.83 |
| 8&9 | I (Ile) and R (Arg) | 31.25 |

**Table** **S3** Screening thresholds for three physicochemical properties.

| Physicochemical properties | Limit |
| --- | --- |
| Net Charge | ∈ [2, 5] |
| GRAVY | ∈ [−0.1, 0.1] |
| Wimley-White hydrophobicity | ∈ [−2.8, −0.18] |

**Table S4** Model runtime environment and hyperparameter settings.

| Operating system | Windows 10 Professional 19045.4780 |
| --- | --- |
| CPU | AMD Ryzen 9 7950X 16-Core Processor 4.50 GHz |
| RAM | USCORSAIR Revenge 16G 5600 MHz |
| GPU | NVIDIA GeForce RTX 4070Ti 12G |
| CUDA version | 12.2 |
| Programming language | Python 3.8 |
| Deep learning framework | Pytorch (Torch 1.12.0, Torchvision 0.13.0) |
| Learning_rate | 0.00001 |
| Batch_size | 256 |
| Pretrained_model | esm2_t30_150M_UR50D |

**Table S5** MICs of 49 c_AFPs against *C. albicans*.

| Sequence | Number^a)^ | Peptide^b)^ | Prediction Value | MIC (μg/mL) ^c)^ |
| --- | --- | --- | --- | --- |
|  |  |  |  | *C.albicans* |
| RRWRIVLIRIAR | 364 | AFP-1 | 0.9017 | 15.63 |
| GLLRIRLIRKAR | 5683 | AFP-2 | 0.9046 | 31.25 |
| RRWRIVVIRVRA | 336 | AFP-3 | 0.9107 | 31.25 |
| KRWRIILIRIAR | 6412 | AFP-4 | 0.9352 | 31.25 |
| GLLRIIKIRKRA | 5826 | AFP-5 | 0.9295 | 31.25 |
| KRWRIIVIRIAR | 6376 | AFP-6 | 0.9472 | 62.50 |
| GLWRIIKIRIRK | 5474 | AFP-7 | 0.9007 | 15.63 |
| RLWRIVVIRRAR | 1318 | AFP-8 | 0.9597 | 7.81 |
| GLLRIRKIRKLA | 5724 | AFP-9 | 0.9300 | 125 |
| RLWRIVLIRRAK | 1355 | AFP-10 | 0.9767 | 7.81 |
| RLWRIVLIRKAR | 1363 | AFP-11 | 0.9685 | 3.91 |
| RLWRIRKIRVLA | 1494 | AFP-12 | 0.9312 | 31.25 |
| VRWRIRLIRKLA | 2448 | AFP-13 | 0.9072 | 7.81 |
| VLWRIRLIRKRA | 3414 | AFP-14 | 0.9226 | 31.25 |
| VLWRIRLIRKAR | 3415 | AFP-15 | 0.9188 | 31.25 |
| KLWRIRVIRRLA | 7263 | AFP-16 | 0.9245 | 15.63 |
| VRWRIVVIRKRA | 2298 | AFP-17 | 0.9284 | 31.25 |
| KRWRIIKIRVLA | 6462 | AFP-18 | 0.9239 | 31.25 |
| KLWRIVKIRIRA | 7203 | AFP-19 | 0.9487 | 31.25 |
| KLWRIIVIRKRA | 7374 | AFP-20 | 0.9486 | 31.25 |
| KRWRIVLIRIAR | 6196 | AFP-21 | 0.9524 | 125 |
| KLWRIVKIRVAR | 7213 | AFP-22 | 0.9336 | 15.63 |
| RRWRIILIRIRA | 579 | AFP-23 | 0.9209 | 31.25 |
| RLWRIVKIRVRA | 1380 | AFP-24 | 0.9492 | 15.63 |
| RLWRIRVIRVAK | 1418 | AFP-25 | 0.9645 | 15.63 |
| VRWRIVLIRKAR | 2335 | AFP-26 | 0.9057 | 15.63 |
| VLWRIRVIRRAK | 3371 | AFP-27 | 0.9249 | 31.25 |
| VLWRIRKIRVRA | 3432 | AFP-28 | 0.9225 | 31.25 |
| KRWRIVKIRVLA | 6246 | AFP-29 | 0.9028 | 15.63 |
| GLARIVLIRRRK | 4916 | AFP-30 | 0.9195 | 31.25 |
| GLLRIVKIRRRA | 5601 | AFP-31 | 0.9291 | 62.50 |
| GLLRIRVIRRAK | 5639 | AFP-32 | 0.9408 | 62.50 |
| KRWRIVLIRIRA | 6195 | AFP-33 | 0.9463 | 62.50 |
| RRWRIIKIRILA | 621 | AFP-34 | 0.9199 | 125 |
| KRWRIVLIRVAR | 6205 | AFP-35 | 0.9606 | 15.63 |
| KLWRIRVIRVRA | 7248 | AFP-36 | 0.9400 | 15.63 |
| RRWRIIVIRVRA | 552 | AFP-37 | 0.9223 | 62.50 |
| KLWRIILIRKRA | 7410 | AFP-38 | 0.9373 | 62.50 |
| VLWRIRKIRKLA | 3456 | AFP-39 | 0.9401 | 62.50 |
| GLARIRVIRKLR | 5002 | AFP-40 | 0.9245 | 62.50 |
| KRWRIVLIRKLA | 6228 | AFP-41 | 0.9333 | 31.25 |
| KLWRIVLIRRAK | 7187 | AFP-42 | 0.9475 | 125 |
| RRWRIVLIRIAK | 365 | AFP-43 | 0.9462 | 15.63 |
| RRWRIVKIRILA | 405 | AFP-44 | 0.9271 | 125 |
| RRWRIIKIRVLA | 630 | AFP-45 | 0.9384 | 62.50 |
| RLWRIVKIRIRA | 1371 | AFP-46 | 0.9288 | 15.63 |
| RLWRIIVIRKRA | 1542 | AFP-47 | 0.9574 | 62.50 |
| RLWRIIVIRKAR | 1543 | AFP-48 | 0.9672 | 15.63 |
| VRWRIILIRKAR | 2551 | AFP-49 | 0.9212 | 15.63 |

a) According to the number of the sequence when it was generated, b) The final selected name, c) The MIC listed is the concentration of the drug in ungrown fungus wells measured in 96-well plates by the microdilution method.

**Table S6** MICs of de novo designed tridecapeptides against fungal pathogens.

| Sequence | Peptide | MIC_100_ (μg/mL) ^a)^ | | |
| --- | --- | --- | --- | --- |
|  |  | *C. albicans* | *C. tropicalis* | *C. parapsilosis* |
| FLPKIGKALKHLF | AFP-T1 | 15.63 | 3.91 | 7.81 |
| FLPHLGKAIKKLF | AFP-T2 | 15.63 | 3.91 | 7.81 |
| FLPHLGKALKHLF | AFP-T3 | 7.81 | 3.91 | 7.81 |
| FLPKLGKAIKHLF | AFP-T4 | 15.63 | 3.91 | 7.81 |
| FLPHIGKALKKLF | AFP-T5 | 15.63 | 3.91 | 7.81 |
| FLPKLGAKLKRLL | AFP-T6 | 31.25 | 1.95 | 15.63 |
| FLPKVGKAIKRLF | AFP-T7 | 15.63 | 3.91 | 15.63 |
| FLSKVGKALKRLF | AFP-T8 | 15.63 | 3.91 | 3.91 |
| FLPHLGKALKKLF | AFP-T9 | 15.63 | 3.91 | 3.91 |
| FLPKLGKAIKKLF | AFP-T10 | 15.63 | 3.91 | 7.81 |
| FLPKVGKALKRLL | AFP-T11 | 15.63 | 3.91 | 31.25 |
| FLPKIGKAIKRLF | AFP-T12 | 7.81 | 1.95 | 7.81 |
| FLPKLGKALKKLL | AFP-T13 | 15.63 | 1.95 | 7.81 |
| FLGKVGKALKRLF | AFP-T14 | 15.63 | 1.95 | 1.95 |
| FLPKLGKALKHLF | AFP-T15 | 15.63 | 1.95 | 3.91 |
| FLPKVGKAIKKLF | AFP-T16 | 31.25 | 3.91 | 31.25 |
| FLPKLGKAIKRLL | AFP-T17 | 15.63 | 1.95 | 7.81 |
| FLPKIGKALKKLF | AFP-T18 | 15.63 | 3.91 | 3.91 |
| FLPHLGKAIKRLF | AFP-T19 | 15.63 | 3.91 | 7.81 |
| FLPKVGKALKHLF | AFP-T20 | 15.63 | 3.91 | 7.81 |
| FLPKVGKALKRLF | AFP-T21 | 7.81 | 1.95 | 7.81 |
| FLPKLGKALKKLF | AFP-T22 | 3.91 | 1.95 | 3.91 |
| FLPKLGKAIKRLF | AFP-T23 | 15.63 | 1.95 | 7.81 |
| FLPKIGKALKRLF | AFP-T24 | 15.63 | 1.95 | 1.95 |
| FLPKVGKALKKLF | AFP-T25 | 15.63 | 1.95 | 7.81 |
| FLPHVGKALKRLF | AFP-T26 | 15.63 | 3.91 | 15.63 |
| FLPHIGKALKRLF | AFP-T27 | 15.63 | 3.91 | 7.81 |
| FLPHVGKALKRLL | AFP-T28 | 31.25 | 3.91 | 15.63 |
| FLPKIGKALKRLL | AFP-T29 | 31.25 | 1.95 | 15.63 |
| FLSKLGKALKRLL | AFP-T30 | 15.63 | 3.91 | 7.81 |
| FLPKIGKAIKKLF | AFP-T31 | 15.63 | 3.91 | 7.81 |
| FLPKVGKALKKLL | AFP-T32 | 31.25 | 3.91 | 31.25 |
| FLPHVGKAIKRLF | AFP-T33 | 15.63 | 1.95 | 3.91 |
| FLPHVGKAIKRLF | AFP-T34 | 15.63 | 3.91 | 15.63 |
| FLPHLGKALKRLL | AFP-T35 | 15.63 | 1.95 | 7.81 |
| FLSKVGKALKKLF | AFP-T36 | 31.25 | 31.25 | 62.50 |
| FLSHVGKALKRLF | AFP-T37 | 15.63 | 1.95 | 3.91 |

a) The MIC_100_ is the concentration of the drug in ungrown fungus wells measured in 96-well plates by the microdilution method.

**Table S7** Detailed distribution of the datasets.

|  | Train | Validation | Test |
| --- | --- | --- | --- |
| AFPs | 2753 | 688 | 688 |
| non-AFPs | 2753 | 688 | 688 |
| Source | Deep-AFPpred | Deep-AFPpred | APD3 |

**Data S1. (separate file)**

Data S1.xlsx
